# Spirodelae Herba ethanol extract attenuates neurotoxicity in hippocampal cells and improves scopolamine-induced cognitive impairment in mice

**DOI:** 10.3389/fphar.2025.1638068

**Published:** 2025-08-28

**Authors:** Yun Hee Jeong, YeonGyun Jung, Wei Li, Hye Jin Yang, You-Chang Oh, Jong-Sup Bae

**Affiliations:** ^1^ Korean Medicine (KM)-Application Center, Korea Institute of Oriental Medicine, Daegu, Republic of Korea; ^2^ Burn Institute, Department of Rehabilitation Medicine, Hangang Sacred Heart Hospital, Hallym University College of Medicine, Seoul, Republic of Korea; ^3^ College of Pharmacy, CMRI, Research Institute of Pharmaceutical Sciences, Kyungpook National University, Daegu, Republic of Korea

**Keywords:** Spirodelae Herba, neuronal protection, antioxidant, neuroinflammation, cognitive impairment, microbiota–gut–brain axis

## Abstract

**Background:**

Spirodelae Herba (SH) is an herb that has been used in traditional medicine in East Asia. Whereas its anti-inflammatory, anti-allergic, and antioxidant activities have recently been demonstrated, the effects of SH ethanol extract (SHE) on neurotoxicity in hippocampal neurons, neuroinflammation in microglia, and cognitive impairment in mice have not been studied.

**Methods:**

In this study, we explored the protective effect of SHE on neurotoxicity related to oxidative stress and the related molecular mechanisms in a hippocampal cell model. We also examined the inhibitory effect of SHE on neuroinflammation and its related mechanisms in endotoxin-stimulated microglia. We also explored the ameliorative effect of SHE on cognitive impairment in mice through behavioral tests and examined histopathological changes in the hippocampus and cortex using Nissl staining. In addition, we conducted a comprehensive analysis of the related mechanisms, including the microbiota–gut–brain axis.

**Results:**

SHE inhibited glutamate-induced neurotoxicity in HT22 cells and induced changes in related mechanisms. SHE effectively inhibited lipopolysaccharide-induced neuroinflammation in BV2 cells and regulated the activation of related mechanisms. In addition, SHE administration significantly alleviated scopolamine (SCO)-induced decreases in memory and learning ability in mice. SHE suppressed damage to hippocampal neurons in the mice’s brain and significantly increased the expression of the brain-derived neurotrophic factor and its related pathway proteins in hippocampal tissue. Furthermore, microbiome analysis revealed that SHE administration normalized SCO-induced gut microbiota imbalance (dysbiosis). These findings indicate that the cognitive improvement effects of SHE may be mediated through the modulation of the gut microbiota composition and the microbiota–gut–brain axis.

**Conclusion:**

The results of this study demonstrate the neuroprotective and anti-neuroinflammatory effects of SHE and its strong potential as a preventive and therapeutic agent for cognitive impairment.

## 1 Introduction

Alzheimer’s disease (AD) is a type of dementia that is a complex degenerative brain disorder that causes mental decline, including memory loss and learning dysfunction (Xian et al., 2015). As societies age worldwide, the proportion of patients with AD gradually increases. More than fifty million people are currently affected by AD worldwide, with nearly ten million new cases occurring each year. It is estimated that by 2050, the number of dementia patients worldwide will exceed 152 million, which is triple the current number (Cummings et al., 2018). The pathophysiological causes of AD include amyloid protein deposition, neurofibrillary tangles, synaptic and neuronal loss, and neuroinflammation (Gulyaeva et al., 2017). Currently, the main treatment medications for AD are donepezil, galantamine, and rivastigmine, which activate the cholinergic neurotransmitter system by increasing acetylcholine (Nwidu et al., 2017). However, these drugs cause several side effects, including nausea, vomiting, headache, and anorexia (Musial et al., 2007). Thus, various studies have focused on investigating the effectiveness of anti-amnesic or anti-dementia agents in traditional herbal medicine in treating various neurodegenerative diseases, including AD.

Oxidative stress is a major cause of neuronal cell death, which is a major feature of AD (Tan et al., 2013). Glutamate is a well-known excitatory neurotransmitter, but excessive glutamate secretion can cause neuronal damage due to oxidative stress (Kim et al., 2019). High concentrations of glutamate induce excessive production of reactive oxygen species (ROS), which leads to mitochondrial dysfunction and cell damage; so, controlling ROS production is an effective way to treat or prevent neurodegenerative diseases by alleviating the risk of oxidative stress (Baillet et al., 2010). Nuclear factor-E2-related factor 2 (Nrf-2) is a transcription factor that regulates the expression of many antioxidant enzymes in the central nervous system, such as NAD(P)H quinone oxidoreductase 1 (NQO1) and heme oxidase (HO)-1 (Cheng et al., 2013). Many studies have demonstrated that the Nrf-2 signaling pathway protects neurons from oxidative damage, and the activation of this pathway exerts a neuroprotective effect against amyloid beta toxicity (Rojo et al., 2017).

The extracellular signal-regulated kinase (ERK) and cAMP response element-binding protein (CREB) signaling molecules are highly correlated with memory storage and formation and are activated by acetylcholine receptors (Kelleher et al., 2004; Li et al., 2017). ERK, a signaling molecule in the hippocampus, activates CREB, a protein related to the development of memory formation and the stabilization of new memories (Adams and Sweatt, 2002). CREB acts as a transcription factor that binds to the promotor region of various neuronal genes involved in memory formation and synaptic plasticity. Brain-derived neurotrophic factor (BDNF) is important for the survival of neurons and mediates synaptic plasticity and cognitive processes (Tyler et al., 2002). In addition, the phosphoinositide 3-kinases (PI3K)/protein kinase B (Akt) signaling pathway is also known to be closely related to synaptic plasticity, amalgamation of recognition memory, and long-term potentiation (LTP) formation (Peltier et al., 2007). Glycogen synthase kinase (GSK)-3β is a downstream intermediate of Akt/PI3K, which is also a key modulator of neurogenesis and synaptic plasticity (Zhang et al., 2016). Thus, the activation of the ERK/CREB/BDNF and the Akt/PI3K/GSK-3β pathways in the brain may help ameliorate cognitive performance and the prevention of neurodegenerative diseases.

Accumulated data from several previous studies indicate that changes in the gut microbiome composition contribute to cognitive dysfunction and behavior (Möhle et al., 2016). Gut microbiota consists of over 1,000 species of bacteria that play a fundamental role in nutrition acquisition, gastrointestinal physiology, energy regulation, and brain function (Buffington et al., 2016; Sharon et al., 2016). Imbalances in the gut microbiota are closely related to causing neuropsychiatric disorders (Hsiao et al., 2013). Recent studies have shown that learning and memory function in mice are linked to diet-induced changes in gut bacteria (Li et al., 2009). Thus, the regulation of the gut microbiota might act as a potential target for improving learning and memory declines and related disorders (Collins et al., 2012).

Spirodelae Herba (SH) is the dried whole plant of *Spirodela polyrrhiza* Schleider (Lemnaceae) and is distributed widely in most regions of Korea, Japan, and China. It has traditionally been used to treat edema, fever, and inflammation-related conditions. Recent studies have shown the anti-inflammatory, anti-allergic, and antioxidant activities of SH, including the inhibition of pro-inflammatory cytokine secretion, mast cell degranulation, and ROS production (Han and Jang, 2016; Shyni et al., 2015). However, the effects of SH ethanol extract (SHE) on oxidative stress-induced neuronal damage, endotoxin-induced neuroinflammation, and scopolamine (SCO)-induced cognitive dysfunction have not been studied yet. In this study, we explored the neuroprotective effects of SHE on HT22 cells exposed to glutamate and the modulatory effects of SHE on microglial BV2-mediated neuroinflammation induced by endotoxin lipopolysaccharide (LPS). In addition, we evaluated the ameliorative effects of SHE on SCO-induced cognitive dysfunction in C57BL/6 mice. We evaluated the role of the microbiome–gut–brain axis in mediating the effects of SHE on ameliorating cognitive impairment by multi-omics analysis of the mouse gut microbiome. Finally, the phytochemical components of SHE were analyzed by high-performance liquid chromatography (HPLC). Furthermore, the correlations between the physiological activity of SHE and its components were investigated.

## 2 Materials and methods

### 2.1 Plant materials

Dried SH was acquired from Yeongcheon Hyundai Herbal Supplier (Yeongcheon, Korea) and confirmed by Professor Ki Hwan Bae, College of Pharmacy, Chungnam National University (Daejeon, Korea). All voucher specimens were deposited in an herbal bank at the KM Application Center, Korea Institute of Oriental Medicine (voucher number: E252). A total of 50.0 g of SH was extracted with 300 mL of a solvent consisting of 70% ethanol and 30% distilled water (DW) at 40 °C and 100 rpm in a shaking incubator for 24 h. The extract was filtered through a filter paper and concentrated in a rotary vacuum evaporator (Buchi, Tokyo, Japan) to remove residual ethanol. It was then freeze-dried to produce SHE powder and stored at −20 °C until use.

### 2.2 Cell culture

Dr. Younghoon Go of the Korea Institute of Oriental Medicine (Daegu, Korea) provided the HT22 cells used in this study. Cells were maintained in complete Dulbecco’s Modified Eagle Medium (DMEM) supplemented with 10% heat-inactivated fetal bovine serum (FBS) and 1% antibiotics at 37 °C in a 5% CO_2_ atmosphere during the experiments. DMEM was purchased from Welgene (Gyeongsan, Korea), and FBS and the antibiotics were purchased from HyClone (Logan, UT, United States).

### 2.3 Measurement of cell viability

HT22 cells were incubated in 96-well culture plates (5 × 10^3^ cells/well) and then subsequently exposed to 5 mM glutamate with or without SHE (10, 20, or 30 μg/mL) or its six main components (isoorientin, orientin, vitexin, cynaroside, cosmosiin, and luteolin). After 24 h, Cell Counting Kit (CCK) solution (10 μL) was added to each well and incubated for 1 more hour. The viability of HT22 cells was determined by optical density at 450 nm using a microplate reader (Molecular Devices, San Jose, CA, United States). The CCK was purchased from Dojindo (Kumamoto, Japan).

### 2.4 Determination of lactate dehydrogenase levels

Lactate dehydrogenase (LDH) release in the culture medium was analyzed using an LDH assay kit according to the manufacturer’s standard protocol. Cells were cultured in 96-well culture plates, pretreated with three concentrations of SHE or its six main components for 2 h, and then exposed to 5 mM glutamate. After 24 h, the cells were centrifuged, and a portion of the supernatant was collected and incubated with the reaction solution. After an additional 30 min of incubation, LDH activity was evaluated at the wavelength of 450 nm by a microplate reader.

### 2.5 Intracellular ROS determination

2,7-Dichlorohydrofluorescein diacetate (H_2_DCFDA) was used to detect intracellular ROS. HT22 cells were seeded at 5 × 10^3^ cells/well in 96-well culture plates. After 18 h, they were pretreated with SHE, cynaroside, or luteolin for 2 h, and then 5 mM glutamate was added to the wells. A total of 20 µM H_2_DCFDA was added to each well for 30 m under dark conditions, and the stained cells were washed three times with cold phosphate-buffered saline (PBS). Fluorescence intensity was estimated using a microplate reader at an excitation wavelength of 485 nm and an emission wavelength of 525 nm, and fluorescence images were captured using a fluorescence microscope (Eclipse Ti, Nikon, Tokyo, Japan). H_2_DCFDA was purchased from Invitrogen (Carlsbad, CA, United States).

### 2.6 Assessment of cell death by flow cytometry

Neuronal apoptosis of HT22 cells was measured and quantified using the FITC Annexin V Apoptosis Detection Kit I (BD Biosciences) according to the standard procedures. HT22 cells were seeded at 5 × 10^5^ cells/well in 6-well culture plates and pretreated with SHE for 2 h and then exposed to 5 mM glutamate for another 24 h. These cells were collected by trypsinization and washed with cold PBS twice. After washing, cells suspended in 500 μL binding buffer were stained with 5 μL propidium iodide (PI) and FITC-conjugated annexin V for 15 min at room temperature (RT) in the dark. These cells were then evaluated on a flow cytometer (FACSCalibur, BD Biosciences).

### 2.7 Immunoblotting analysis

Mouse brain tissues and HT22 whole cells were lysed with radioimmunoprecipitation assay lysis buffer (Millipore, Burlington, MA, United States) containing protease and phosphatase inhibitors (Roche, Basel, Switzerland) and incubated on ice for 1 h before centrifugation to obtain protein lysates. The protein concentration of each sample was standardized using a BCA Kit (Thermo Fisher Scientific), and equal amounts of protein (20 µg) were separated by sodium dodecyl sulfate–polyacrylamide gel electrophoresis. The proteins were blotted onto polyvinylidene fluoride membranes and blocked with a solution containing 3% bovine serum albumin and Tween 20 in tris-buffered saline. The membranes were then incubated overnight at 4 °C with each primary antibody (1:1,000 dilution) and washed four times. The membranes were incubated with the appropriate secondary antibodies diluted at 1:5,000 for 1 h at RT and analyzed using a ChemiDoc™ Touch Imaging System (Bio-Rad, Hercules, CA, United States). Each protein band was normalized to its housekeeping protein and analyzed using ImageJ software (version 1.53e; National Institutes of Health, Bethesda, MD, United States). Table 1 details the primary and secondary antibodies used for the Western blot analysis.

**TABLE 1:** Primary and secondary antibodies used for Western blot analysis.

Antibody	Corporation	Product no.	RRID	Dilution rate
Nrf-2	Cell Signaling	#12721	AB_2715528	1:1,000
Lamin B1	Cell Signaling	#13435	AB_2737428	1:1,000
HO-1	Cell Signaling	#82206	AB_2799989	1:1,000
NQO1	Santa Cruz	#SC-32793	AB_628036	1:1,000
β-Actin	Cell Signaling	#4970	AB_2223172	1:1,000
P-ERK	Cell Signaling	#4377	AB_331775	1:1,000
ERK	Cell Signaling	#9102	AB_330744	1:1,000
P-CREB	Cell Signaling	#9191	AB_331606	1:1,000
CREB	Cell Signaling	#9197	AB_331277	1:1,000
BDNF	Cell Signaling	#47808	AB_2894709	1:1,000
NF-κB p65	Cell Signaling	#8242	AB_10859369	1:1,000
P-p38	Cell Signaling	#9211	AB_331641	1:1,000
P38	Cell Signaling	#9212	AB_330713	1:1,000
P-JNK	Cell Signaling	#9251	AB_331659	1:1,000
JNK	Cell Signaling	#9252	AB_2250373	1:1,000
P-PI3K	Cell Signaling	#17366	AB_2895293	1:1,000
PI3K	Cell Signaling	#4257	AB_659889	1:1,000
P-Akt	Cell Signaling	#4060	AB_2315049	1:1,000
Akt	Cell Signaling	#4691	AB_915783	1:1,000
P-GSK-3β	Cell Signaling	#9336	AB_331405	1:1,000
GSK-3β	Cell Signaling	#9315	AB_490890	1:1,000
2nd anti-mouse	Cell Signaling	#7076	AB_330924	1:5,000
2nd anti-rabbit	Cell Signaling	#7074	AB_2099233	1:5,000

### 2.8 Analysis of nitric oxide (NO) secretion

Nitric oxide (NO) secretion was estimated by measuring the nitrite concentration in the BV2 cell culture medium using Griess reagent. BV2 cells cultured for 18 h in 96-well culture plates were treated with SHE (10, 50, or 100 μg/mL) and exposed to 100 ng/mL LPS for 1 h and later for 24 h. Then, the same amount of Griess reagent as the culture medium was added and incubated for 5 min at RT, and the absorbance was measured at 570 nm using a microplate reader.

### 2.9 Enzyme-linked immunosorbent assay (ELISA) for cytokine measurement

Inflammatory cytokine levels in BV2 cell culture medium were measured using an ELISA Kit (Invitrogen, Carlsbad, CA, United States) according to the manufacturer’s protocol. BV2 cells cultured for 18 h in 24-well culture plates were pretreated with SHE for 1 h and stimulated with LPS for another 6 h. After that, the supernatant was obtained by removing the cell debris through centrifugation (15,000 *g*), and the cytokine levels were measured.

### 2.10 Animals and treatment

C57BL/6 mice (4 weeks old) were obtained from Samtako Bio Korea (Osan, Korea) and used for *in vivo* experiments. Male C57BL/6 mice were housed in a specific pathogen-free facility under controlled conditions (12-h light/dark cycle at 22 °C ± 2 °C), and food and water were provided *ad libitum*. All mice were allowed to acclimate to the new housing environment for 1 week before the experiment and randomly divided into four groups of eight mice each: normal (vehicle), SCO (SCO + vehicle), SHE 100 (SCO + SHE 100 mg/kg), and SHE 200 (SCO + SHE 200 mg/kg). SCO was acquired from Sigma-Aldrich (St. Louis, MO, United States) and used to induce cognitive impairment in mice. All mice were orally administered the vehicle or SHE once daily for 4 weeks, and 1 mg/kg of SCO was injected once daily to the mice in the SCO, SHE 100, and SHE 200 groups during the last week. All animal experiments were approved by the Animal Care and Use Committee of the Korea Institute of Oriental Medicine (reference number #D-22-045) and followed the relevant guidelines.

### 2.11 Morris water maze tests

The water maze pool was divided equally into four quadrants, with a platform hidden approximately 1 cm below the water, as described previously (Morris, 1984). The Morris water maze (MWM) test was started on the 28th day after SHE administration to evaluate the spatial learning memory ability of the mice. Each trial lasted 60 s until the mouse found the hidden platform and climbed up on it. The mice underwent an acquisition trial for 6 days. The MWM test was analyzed by a video camera equipped with the SMART video tracking system (Panlab, Barcelona, Spain).

### 2.12 Passive avoidance test

The passive avoidance (PA) test was performed to measure the working memory of the mice using the Shuttle Box Avoidance Basic Test Package (Med Associates Inc., Fairfax, VT, United States). This apparatus consists of two compartments connected to each other by a guillotine door. One of them is illuminated, and the other is a dark compartment with an electric bar mounted on the floor. On the training day, each mouse was placed in the illuminated compartment, and after a 30-s acclimation period, the guillotine door was opened, and when the mice moved to the dark chamber, a 0.2-mA electric foot shock (5 s) was delivered after a 2-s delay. The test procedure was the same in the test conducted 24 h later, but no electric shock was delivered when moving to the dark chamber. The latency to move to the dark compartment was recorded in all tests, up to a maximum of 180 s.

### 2.13 Nissl staining

After the mice were sacrificed, brain tissue was collected and post-fixed in a 4% paraformaldehyde solution for 24 h. Specimens were dehydrated, immersed in paraffin, and then sliced into 5-*µ*m-thick sections at RT for later use. Tissue sections were immersed in cresyl violet solution (0.5%) for 30 min, rinsed with DW, dehydrated with different ethanol concentrations, cleared using xylene, and mounted on glass coverslips (Chen et al., 2018). Images were visualized using a microscope.

### 2.14 Gut microbiome analysis

Fresh fecal samples were collected from all the mice in each group, and the collected samples were placed in sterile tubes, immediately frozen in liquid nitrogen, and stored at −80 °C. DNA from each sample was extracted using the QIAamp PowerFecal DNA Isolation Kit (Qiagen), and the quality of the extracted DNA was assessed by electrophoresis and quantified using a Qubit 2.0 Fluorometer (Life Technologies, Carlsbad, CA, United States). 16S rRNA gene sequencing was then performed using the Illumina MiSeq platform at the KNU NGS Core Facility (Kyungpook National University, Daegu, Korea). Data were analyzed by QIIME 2 (version 2022.2) using DADA2 software (Bolyen et al., 2019; Callahan et al., 2016). The taxonomic classification of the representative sequences was determined through taxonomy using the q2-feature-classifier (sklearn) against the Greengenes database (version 13.8) (DeSantis et al., 2006; Pedregosa et al., 2011). Phylogenetic trees, both rooted and unrooted, were generated using the MAFFT, mask, and FastTree protocols (Katoh and Standley, 2013; Price et al., 2010). These trees were subsequently used for diversity analysis. The smallest sample contained 37,376 features. Further analyses were performed using the phyloseq (version 1.32.0) and vegan (version 2.5-6) R packages (McMurdie and Holmes, 2013).

### 2.15 SHE sample preparation for HPLC analysis

All standard solutions contained methanol as the solvent to dissolve the compounds. Initially, standard stock solutions with 100 μg/mL concentration were prepared, followed by serial dilution with methanol to create a range of concentrations for the calibration curve. The SHE sample solution was prepared at 20 mg/mL concentration in methanol. Prior to analysis, all solutions were filtered using 0.45-μm regenerated cellulose membrane syringe filters (Sartorius, Germany). Table 2 details the compounds used for HPLC analysis.

**TABLE 2:** Detailed information of the compounds used for HPLC analysis.

	Compounds	CAS no.	Company	Product no.	Assay (HPLC) (%)
1	Isoorientin	4261-42-1	Sigma-Aldrich	#I1536	≥98
2	Orientin	28608-75-5	Sigma-Aldrich	#O9765	≥97
3	Vitexin	3681-93-4	Sigma-Aldrich	#49513	≥98
4	Cynaroside	5,373-11-5	Sigma-Aldrich	#49968	≥98
5	Cosmosiin	578-74-5	Sigma-Aldrich	#44692	≥97
6	Luteolin	491-70-3	Sigma-Aldrich	#L9283	≥98

### 2.16 Optimization of chromatographic conditions

HPLC analyses were carried out using a Dionex UltiMate 3000 system (Dionex Corp., Sunnyvale, CA, United States), which includes a binary pump, autosampler, column oven, and diode array UV/Vis detector (DAD). The operation of the system and data analysis were managed using Dionex Chromeleon software. Chromatographic separation was achieved using a Luna C18 column (250 mm × 4.6 mm, 5 μm, Phenomenex, Torrance, CA, United States). The column oven was maintained at 30 °C, and detection was performed at a UV wavelength of 320 nm. The mobile phase was composed of water (solvent A) and acetonitrile (solvent B), with gradient elution programmed as follows: a total of 0 min–60 min, with a gradient of 5% B (0 min–5 min), 5%–15% B (5 min–12 min), 15%–35% B (12 min–42 min), and 35%–80% B (42 min–60 min). The flow rate was set to 1.0 mL/min. After each run, the column was re-equilibrated to the initial solvent conditions for 10 min before the next sample injection.

### 2.17 Validation of the analytical HPLC method

Specificity was evaluated by comparing the chromatographic profiles of the standard with those of the sample extract. Linearity was determined by the correlation coefficient (r^2^) of the calibration curve for each compound at concentrations ranging from 15.625 μg/mL to 500 μg/mL. The regression equations were derived using the formula y = ax ± b, where y represents the peak area and x denotes the concentration of the sample.

### 2.18 Statistical analysis

The data values are expressed as the means with standard error of the mean from at least three independent replicate experiments. Statistical significance was measured through one-way analysis of variance and verified using Dunnett’s test after comparing SCO, glutamate, or LPS vs. each treated group. Statistical significance was determined as ^#^ *p* < 0.05 (vs. normal or control); ^*^ *p* < 0.05, ^**^ *p* < 0.01, and ^†^ *p* < 0.001 (vs. SCO, glutamate, or LPS); and ^‡^ *p* < 0.05 (vs. glutamate + SHE 30).

## 3 Results

### 3.1 Mitigating effect of SHE on neurotoxicity in HT22 hippocampal cells exposed to glutamate

**FIGURE 1:**
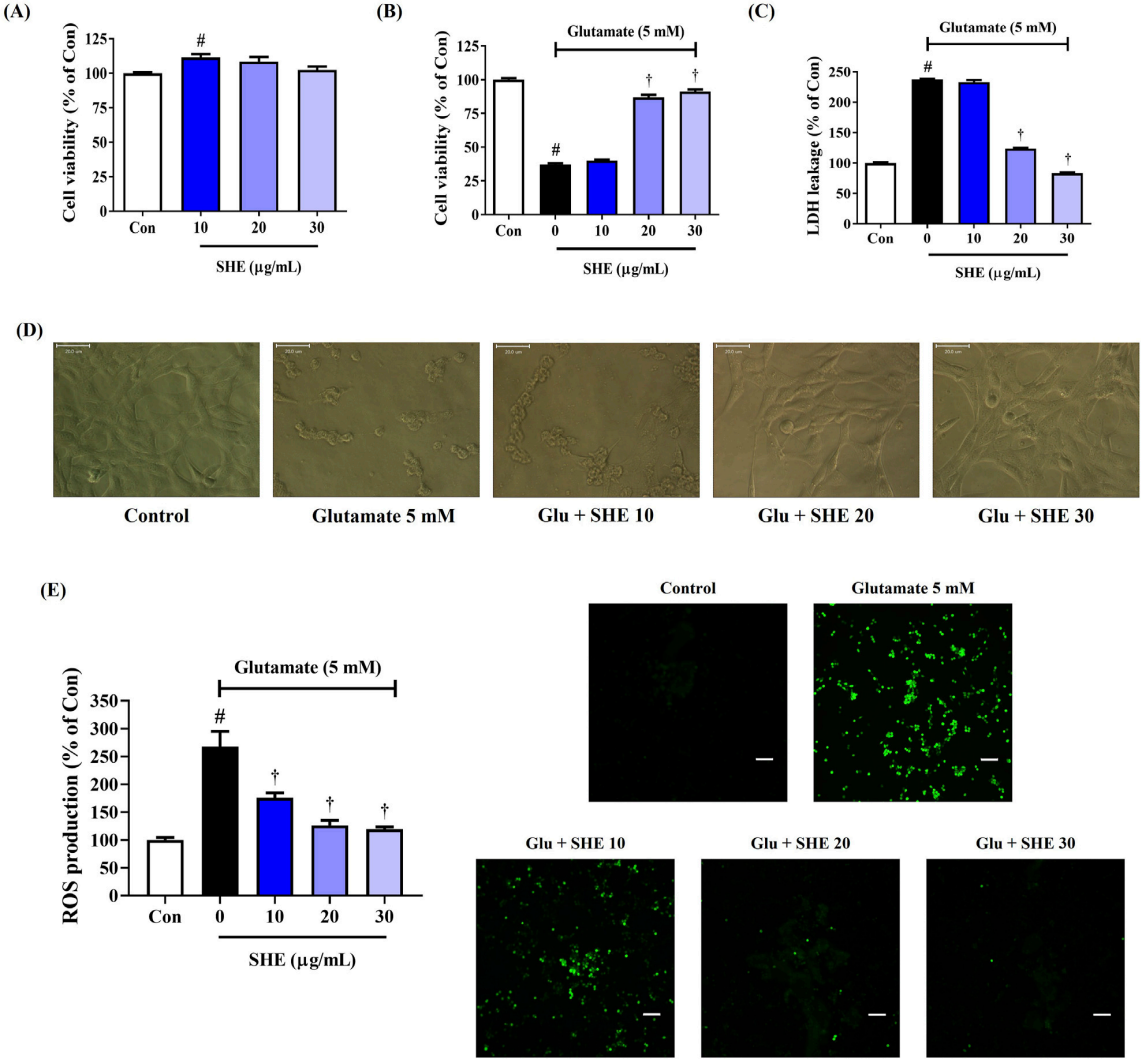
Effects of Spirodelae Herba ethanol extract (SHE) on glutamate-induced **(A–D)** cytotoxicity and **(E)** intracellular reactive oxygen species (ROS) production in HT22 cells. Control cells were incubated with the vehicle alone. All experiments were repeated at least three times, and similar results were obtained. **(A–C, E)** Data are expressed as the mean ± standard error of the mean of six independent experiments. **(D)** Scale bar = 20 μm. **(E)** Scale bar = 200 μm. Con, control; LDH, lactate dehydrogenase; Glu, glutamate. ^#^ *p* < 0.05 (vs. control) and ^†^ *p* < 0.001 (vs. glutamate).

We treated HT22 mouse hippocampal neurons with SHE at different concentrations to investigate its effect on cell viability *in vitro*. The CCK assay showed that SHE treatment alone had no effect on the viability of HT22 cells at up to 30 μg/mL concentration (Figure 1A). Therefore, in subsequent experiments, SHE was administered at doses of 30 μg/mL or less. Next, we measured the effect of SHE on glutamate-exposed neurotoxicity in HT22 cells. An approximately 63% decrease in cell viability was observed after exposure to glutamate (Figure 1B). However, SHE pretreatment effectively improved cell viability in a concentration-dependent manner. Upon pretreatment with 30 μg/mL SHE, cell viability improved to approximately 91% (Figure 1B). Treatment with glutamate increased the leakage of LDH, an indicator of apoptosis, by 225%, whereas pretreatment with SHE at any concentration (10 μg/mL–30 μg/mL) significantly reduced LDH leakage (Figure 1C). This protective effect of SHE against neurotoxicity was also observed against changes in cell morphology. Glutamate treatment induced nuclear condensation and cell shrinkage in HT22 cells, whereas pretreatment with SHE dose-dependently maintained the cell structure and inhibited morphological changes (Figure 1D).

### 3.2 Inhibitory effect of SHE on ROS production in HT22 cells exposed to glutamate

We analyzed ROS accumulation in HT22 cells by fluorescence intensity with an H_2_DCFDA probe to demonstrate the effect of SHE on neuronal oxidative stress caused by glutamate exposure. ROS accumulation notably increased in HT22 cells treated with glutamate. However, pretreatment with SHE significantly and concentration-dependently reduced glutamate-induced intracellular ROS production (Figure 1E).

### 3.3 Mitigating effect of SHE on neuronal cell death induced by glutamate exposure

**FIGURE 2:**
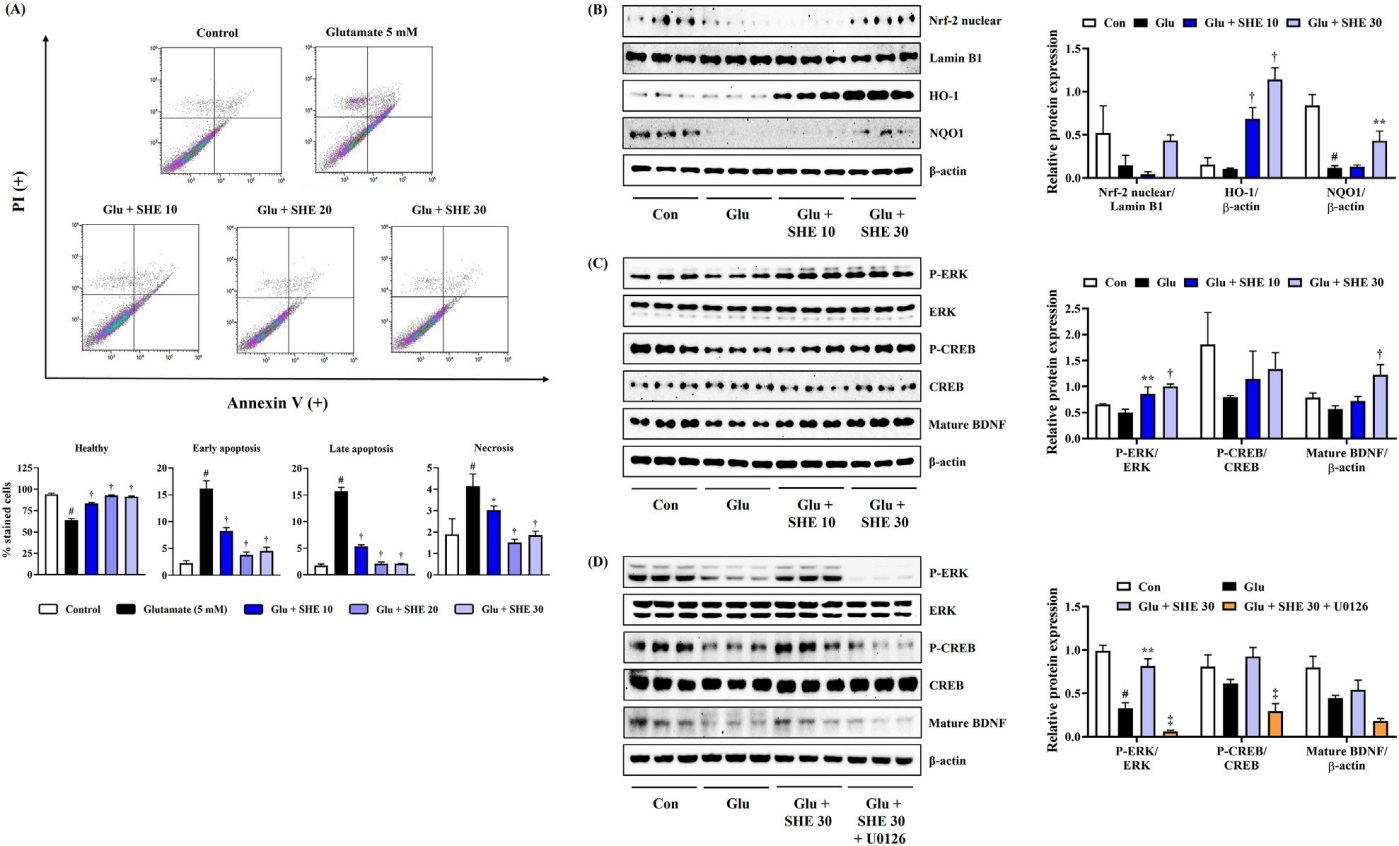
Effects of Spirodelae Herba ethanol extract (SHE) on **(A)** apoptosis, **(B)** Nrf-2 pathway activation, **(C)** ERK/CREB/BDNF activation, and **(D)** ERK/CREB/BDNF activation with U0126 in glutamate-exposed HT22 cells. **(A)** Data are expressed as the mean ± standard error of the mean of six independent experiments. **(B–D)** Histograms show the expression levels of proteins relative to those of the housekeeping protein. Data are expressed as the mean ± standard error of the mean values of three independently obtained protein samples from Western blot analysis. Con, control; Glu, glutamate; Nrf-2, nuclear factor-E2-related factor 2; HO, heme oxygenase; NQO1, NAD(P)H quinone oxidoreductase 1; ERK, extracellular signal-regulated kinase; CREB, cAMP response element-binding protein; BDNF, brain-derived neurotrophic factor; LDH, lactate dehydrogenase. ^#^ *p* < 0.05 (vs. control), ^*^ *p* < 0.05, ^**^ *p* < 0.01, ^†^ *p* < 0.001 (vs. glutamate), and ^‡^ *p* < 0.05 (vs. glutamate + SHE 30).

To explore the activity of SHE on neuronal apoptosis exposed by glutamate treatment, flow cytometric analysis was performed using annexin V–PI double staining on damaged cells. Flow cytometry revealed that glutamate exposure for 24 h notably increased the apoptosis of HT22 cells. However, SHE pretreatment significantly attenuated glutamate-treated neuronal cell death concentration-dependently, and treatment at 30 μg/mL concentration maintained cell numbers similar to that of the non-treated control (Figure 2A).

### 3.4 SHE induces Nrf-2-mediated antioxidant enzyme expression in HT22 hippocampal cells exposed to glutamate

As Nrf-2 activation promotes antioxidant enzyme expression and suppresses oxidative stress, exerting neuroprotective effects, we analyzed the effects of SHE on the nuclear translocation of Nrf-2 and the expression of antioxidant enzyme in HT22 cells. Figure 2B shows that SHE pretreatment increased the nuclear translocation of Nrf-2, effectively induced the expression of HO-1, and significantly restored the expression of NQO1 that was decreased by glutamate. Therefore, SHE induced an increase in antioxidant enzyme levels through the activation of Nrf-2, suggesting that the antioxidant properties contributed to the neuroprotective efficacy of SHE to some extent. Therefore, the activation of the Nrf-2 signaling pathway is thought to be involved in the neuroprotective effect of SHE.

### 3.5 SHE activates the ERK/CREB/BDNF pathway in HT22 hippocampal cells exposed to glutamate

We also analyzed the effects of SHE on ERK/CREB pathway activation and BDNF expression in HT22 cells treated with glutamate. Figure 2C shows that SHE effectively activated ERK and CREB by inducing phosphorylation compared to HT22 cells treated with glutamate alone, suggesting that it induced mature BDNF expression. To further evaluate the role of ERK in the neuroprotective effect of SHE, we evaluated the effect of combined treatment with SHE and MEK inhibitor U0126 (10 μM) on the ERK/CREB/BDNF pathway. As shown in Figure 2D, U0126 significantly reversed the SHE-induced increase in ERK/CREB phosphorylation and BDNF expression in glutamate-stimulated HT22 cells. These results suggest that SHE alleviates glutamate-induced neuronal apoptosis through the activation of the ERK/CREB/BDNF signaling pathway.

### 3.6 Inhibitory effects of SHE on neuroinflammation through regulation of the nuclear factor (NF)-κB/mitogen-activated protein kinase (MAPK) signaling pathway

**FIGURE 3:**
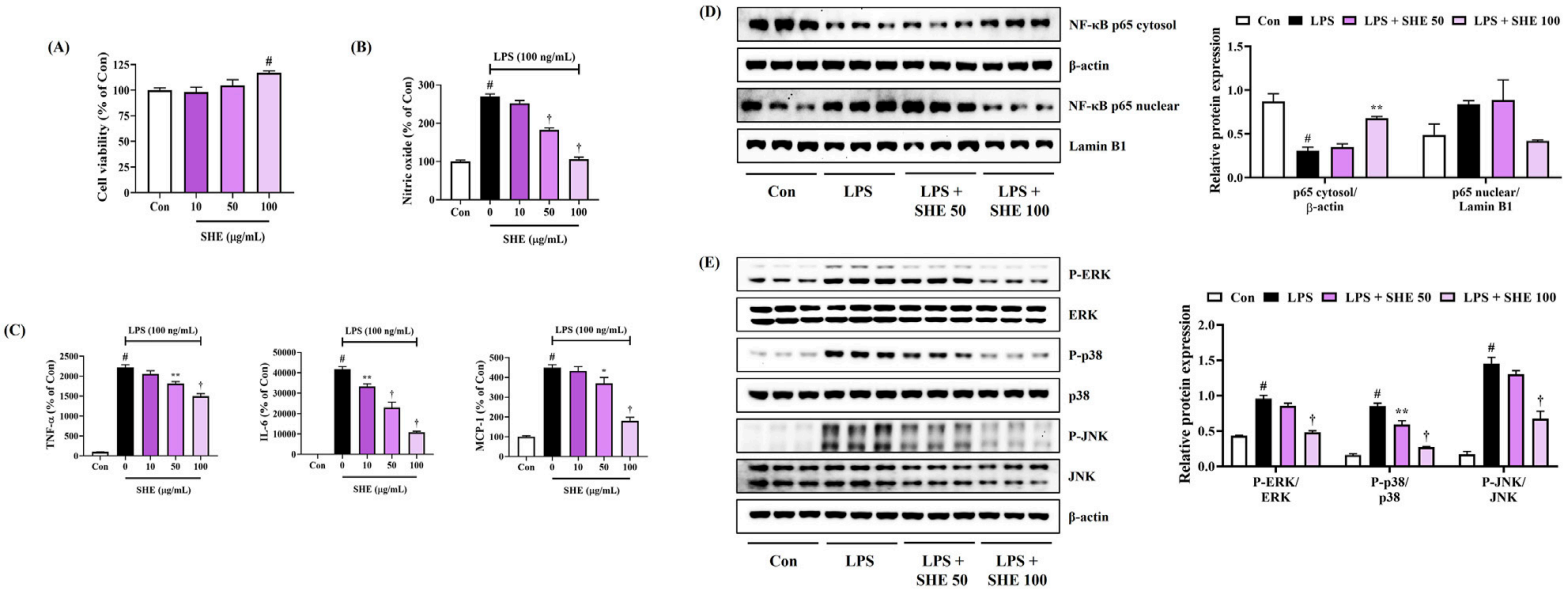
Effects of Spirodelae Herba ethanol extract (SHE) on **(A)** viability, **(B)** secretion of NO, **(C)** production of inflammatory cytokines, and **(D, E)** activation of NF-κB/MAPK pathways in LPS-stimulated BV2 cells. **(A–C)** Data are expressed as the mean ± standard error of the mean of six independent experiments. **(D, E)** Histograms show the expression levels of proteins relative to those of the housekeeping protein. Data are expressed as the mean ± standard error of the mean values of three independently obtained protein samples from Western blot analysis. Con, control; LPS, lipopolysaccharide; TNF, tumor necrosis factor; IL, interleukin; MCP, monocyte chemoattractant protein; NF, nuclear factor; ERK, extracellular signal-regulated kinase; JNK, c-Jun NH_2_-terminal kinase. ^#^ *p* < 0.05 (vs. control), ^*^ *p* < 0.05, ^**^ *p* < 0.01, and ^†^ *p* < 0.001 (vs. LPS).

We first investigated the cytotoxicity of SHE against BV2 microglial cells using the CCK assay. As shown in Figure 3A, SHE treatment did not show cytotoxicity up to a concentration of 100 μg/mL. Therefore, subsequent experiments were performed at concentrations below 100 μg/mL. Next, we measured the production of neuroinflammatory factors to evaluate the anti-neuroinflammatory effect of SHE. Figure 3B shows that SHE treatment markedly inhibited NO secretion in a concentration-dependent manner after LPS stimulation. In addition, the level of inflammatory cytokines, including TNF-α, IL-6, and MCP-1, was effectively suppressed by SHE treatment (Figure 3C).

NF-κB is a key factor that plays an important role in regulating various pro-inflammatory mediators, such as NO and inflammatory cytokines (Roshak et al., 1996). Thus, we examined the effects of SHE on changes in the transcription factor (NF-κB) in LPS-stimulated BV2 cells. The Western blot analysis showed that SHE concentration-dependently inhibited p65 translocation into the nucleus (Figure 3D). In addition, as phosphorylation-activated MAPKs have a crucial role in regulating NF-κB activation, we additionally assessed the inhibitory effects of SHE treatment on the activation of the MAPK pathway. Our data revealed that SHE treatment strongly diminished phosphorylation of all the members of the MAPK family, including ERK, p38, and JNK (Figure 3E). These results suggest that SHE inhibits the expression of pro-inflammatory factors by regulating the NF-κB/MAPK signaling pathway in LPS-stimulated BV2 microglial cells.

### 3.7 Alleviation of SCO-induced decreased spatial memory function in mice by SHE

**FIGURE 4:**
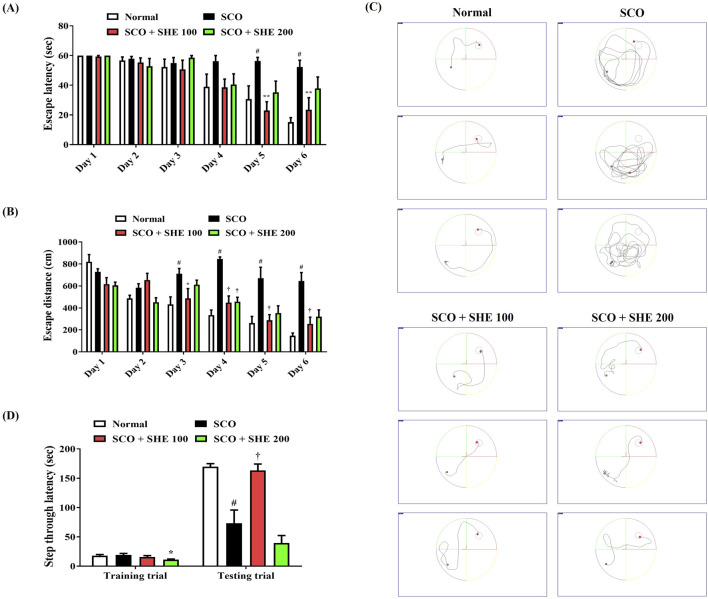
Effects of Spirodelae Herba ethanol extract (SHE) on scopolamine (SCO)-induced cognitive impairment in mice. Spatial memory function in mice was determined by the **(A)** escape latency, **(B)** escape distance, and **(C)** swimming trajectory in the Morris water maze test. **(C)** Images represent the swimming trajectory of mice in each group on day 6. Working memory ability was examined by **(D)** step-through latency in the passive avoidance test. **(A, B, D)** Data are expressed as the mean ± standard error of the mean of eight mice. ^#^ *p* < 0.05 (vs. normal), ^*^ *p* < 0.05, ^**^ *p* < 0.01, and ^†^ *p* < 0.001 (vs. SCO).

We first determined the spatial memory function of mice using the MWM test to explore the effects of SHE on SCO-induced cognitive impairment. As shown in Figures 4A–C, the mice injected with SCO showed poor learning ability to find the hidden platform than the normal group. In contrast, the 100 mg/kg SHE-treated group showed a relatively quick and good learning ability to find the platform. From day 4 to day 6, the average escape latency and distance increased significantly in the SCO group, indicating that intraperitoneal administration of SCO reduced the spatial memory function of the mice. However, 100 mg/kg SHE administration strongly reduced both the latency (Figure 4A) and distance (Figure 4B) of escaping the water maze. On the other hand, administration of 200 mg/kg SHE showed a tendency to decrease both the escape latency and escape distance compared with SCO alone, but the effect was lower than that of 100 mg/kg and was not significant. In addition, the degree of recovery of the spatial memory function according to SHE administration by dose was also well demonstrated in the swimming trajectory on day 6 (Figure 4C).

### 3.8 Improvement of SCO-induced decrease in working memory in mice by SHE

Following the MWM test, a PA test was conducted to explore the influences of SCO and SHE administration on the working memory of mice. Little difference was found between each group in the training trial, but in the testing trial, the step-through latency of mice was significantly decreased by SCO administration (Figure 4D). However, the 100 mg/kg SHE group showed a significantly higher step-through latency than the SCO group, indicating that 100 mg/kg SHE administration improved the mice’s working memory (Figure 4D). In addition, the 200 mg/kg SHE group showed no improvement in working memory at all compared with the SCO group, suggesting that the efficacy of 100 mg/kg SHE was much better than that of 200 mg/kg, as shown in the MWM test (Figure 4D). Therefore, only the 100 mg/kg SHE group sample, which showed a lower dose but better cognitive improvement efficacy, was used in the subsequent *in vivo* analysis tests.

### 3.9 SHE alleviated SCO-induced neuronal injury in the mice brain

**FIGURE 5:**
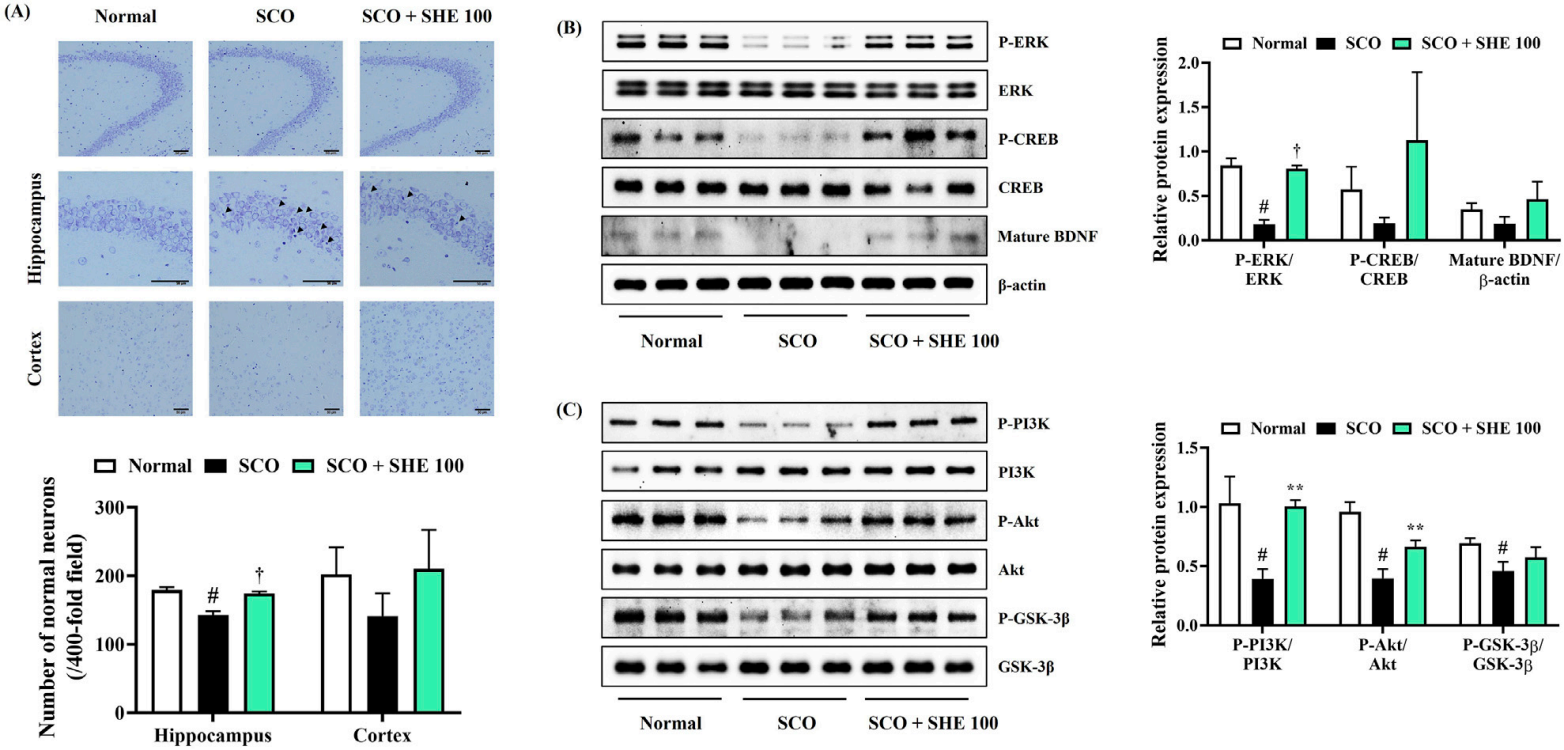
Effects of Spirodelae Herba ethanol extract (SHE) on scopolamine (SCO)-induced **(A)** neuronal damage and depression of the **(B)** ERK/CREB/BDNF and **(C)** PI3K/Akt/GSK-3β signaling pathways in mice brains. **(A)** Scale bars = 50 μm. Data are expressed as the mean ± standard error of the mean of six randomly selected fields. **(B, C)** Histograms show the expression levels of proteins relative to those of the housekeeping protein. Data are expressed as the mean ± standard error of the mean values of three independently obtained protein samples from Western blot analysis. ERK, extracellular signal-regulated kinase; CREB, cAMP response element-binding protein; BDNF, brain-derived neurotrophic factor; PI3K, phosphoinositide 3-kinases; Akt, protein kinase B; GSK, glycogen synthase kinase. ^#^ *p* < 0.05 (vs. normal), ^*^ *p* < 0.05, ^**^ *p* < 0.01, and ^†^ *p* < 0.001 (vs. SCO).

Histopathological changes in brain tissue were analyzed by Nissl staining with 0.5% cresyl violet solution to confirm neuronal damage in the mice brains caused by SCO injection and the effect of SHE administration thereon. Compared with that of normal mice, the hippocampal regions of mice treated with SCO alone appeared irregularly arranged and shrunken, indicating extensive damage, neuronal apoptosis, and the loss of large amounts of Nissl bodies (Figure 5A). Conversely, in the cortex and hippocampus of 100 mg/kg SHE-administered mice, the number of neurons with normal morphology was effectively restored (Figure 5A).

### 3.10 SHE increased ERK, CREB, PI3K, Akt, GSK-3β phosphorylation, and BDNF expression in mice hippocampal tissues

Next, hippocampal tissues were analyzed by immunoblotting to measure the signaling molecules associated with the alleviating effects of SHE on cognitive decline and neuronal damage in mice. The levels of ERK, CREB, PI3K, Akt, GSK-3β phosphorylation, and BDNF expression in mouse hippocampal tissue were significantly reduced by SCO injection. Conversely, the decreased ERK, CREB phosphorylation, and BDNF expression were strongly increased by 100 mg/kg SHE administration (Figure 5B). The activation levels of PI3K and its downstream signaling molecules Akt and GSK-3β, which were reduced by SCO, were also significantly restored by 100 mg/kg SHE administration (Figure 5C).

### 3.11 SHE restored gut microbiome composition in mice

**FIGURE 6:**
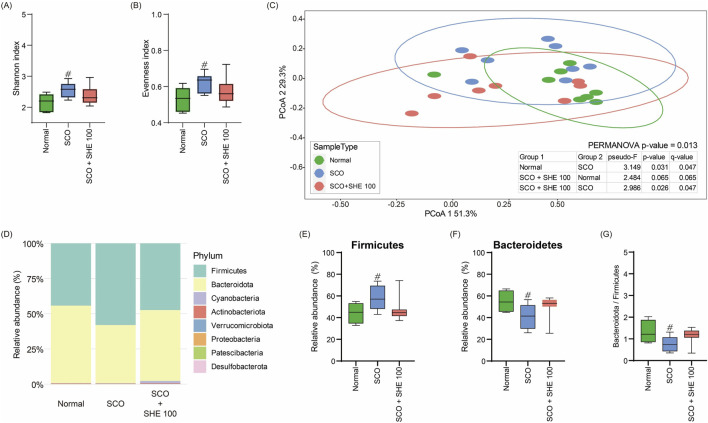
Effects of Spirodelae Herba ethanol extract (SHE) on gut microbiota diversity and composition in the scopolamine (SCO)-induced cognitive impairment mouse model. **(A)** Shannon and **(B)** evenness indices indicating the alpha diversity of gut microbiota. **(C)** Principal coordinates analysis based on Bray–Curtis dissimilarity showing the beta diversity of gut microbiota. **(D)** Relative abundance of major bacterial phyla. **(E)** Relative abundance of Firmicutes. **(F)** Relative abundance of Bacteroidetes. **(G)** Ratio of Bacteroidetes to Firmicutes in the normal, SCO, and SCO + SHE 100 groups. ^#^ *p* < 0.05 (vs. normal).

To examine the effect of SHE on the gut microbiome of SCO-induced cognitive impairment in mice, we performed a comprehensive analysis of microbial diversity and composition. Comparison of the alpha diversity of the gut microbiome of mice showed that the Shannon and evenness indices significantly increased following SCO administration. However, 100 mg/kg SHE administration restored microbial diversity to near normal levels (Figures 6A, B). Principal coordinates analysis revealed a distinct clustering of microbiome profiles between the SCO group and both the normal and 100 mg/kg SHE-administered groups. No significant clustering was observed between the 100 mg/kg SHE-administered and normal groups (Figure 6C). These findings demonstrate that 100 mg/kg SHE restored the microbiome composition altered by SCO administration. Relative abundance analysis indicated changes in microbial composition in the SCO-only group, including a decrease in Bacteroidetes and an increase in Firmicutes. Treatment with 100 mg/kg SHE significantly restored the balance of these bacterial phyla (Figures 6D–G). These results indicate that 100 mg/kg SHE administration not only mitigated cognitive impairment and neuronal damage but also positively modulated the gut microbiome in SCO-induced mice.

### 3.12 Identification of the components of SHE using HPLC–DAD analysis

**FIGURE 7:**
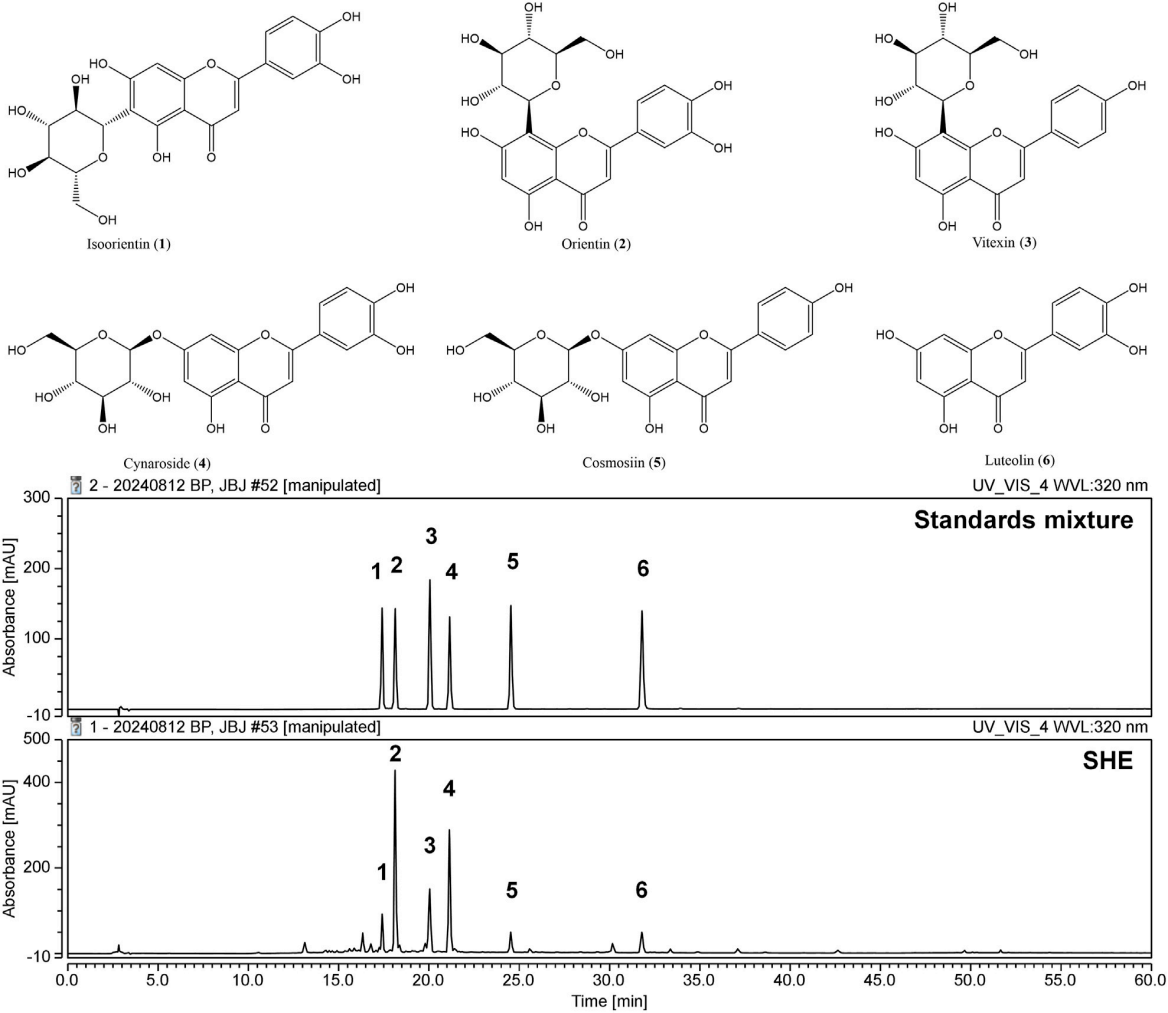
Structures of compounds 1–6 and high-performance liquid chromatograms of the six standard compounds derived from SHE at UV wavelengths of 320 nm. Isoorientin (**1**), orientin (**2**), vitexin (**3**), cynaroside (**4**), cosmosiin (**5**), and luteolin (**6**) were identified.

The primary components of SHE were flavonoids, which are well known for their diverse biological activities. The six key flavonoid compounds identified in the current analysis were evaluated further. The mobile phase used for the separation consisted of acetonitrile and water, which were optimized to achieve clear resolution of the components. A photodiode array detector was used specifically to monitor the absorbance at a wavelength of 320 nm, which is characteristic of flavonoid compounds. The identification of these flavonoids was based on a comparative analysis of their chromatographic retention times (tR) and UV absorption spectra with those of known standards (Figure 7). The retention times for the standards were as follows: isoorientin (compound **1**, tR = 17.41 min), orientin (compound **2**, tR = 18.12 min), vitexin (compound **3**, tR = 20.04 min), cynaroside (compound **4**, tR = 21.13 min), cosmosiin (compound **5**, tR = 24.53 min), and luteolin (compound **6**, tR = 31.78 min). Chromatographic separation achieved excellent selectivity for all six flavonoids, with no significant interferences observed within the 60-min analysis.

The contents of compounds **1**–**6** were determined based on calibration curves created by plotting the peak area against the concentration for each analyte using least-squares regression analysis. Each calibration equation was derived from five concentration levels, ranging from 15.625 μg/mL to 250 μg/mL for isoorientin (**1**), vitexin (**3**), cosmosiin (**5**), and luteolin (**6**), and from 31.25 μg/mL to 250 μg/mL for orientin (**2**) and cynaroside (**4**). The results indicated that the concentrations of the six compounds ranged from 4.94 mg/g to 26.73 mg/g (Table 3). This finding suggests that this validation method is suitable for detecting trace amounts in the extract.

**TABLE 3:** Regression data and contents of the six main components in SHE.

Analytes	Regression equation	*r* ^2^	Content (mg/g)
Isoorientin (**1**)	*y* = 0.2683*x* − 0.3388	0.9999	4.94
Orientin (**2**)	*y* = 0.2656*x* + 0.0279	1	26.73
Vitexin (**3**)	*y* = 0.2605*x* − 0.2952	0.9999	8.19
Cynaroside (**4**)	*y* = 0.2749*x* + 0.2051	1	19.65
Cosmosiin (**5**)	*y* = 0.2176*x* − 0.2735	0.9999	3.05
Luteolin (**6**)	*y* = 0.3987*x* − 0.5806	0.9999	3.18

The successful identification and quantification of these flavonoids established flavonoids as the major chemical components of SHE. Orientin and cynaroside were found at the highest concentrations among the components detected, indicating their prominence in the overall flavonoid profile of SHE. This analysis not only reinforced the significance of flavonoids in SHE but also highlighted the reliability of HPLC–DAD in the precise identification of bioactive components.

### 3.13 Effects of the six main components of SHE on neurotoxicity in HT22 hippocampal cells exposed to glutamate

**FIGURE 8:**
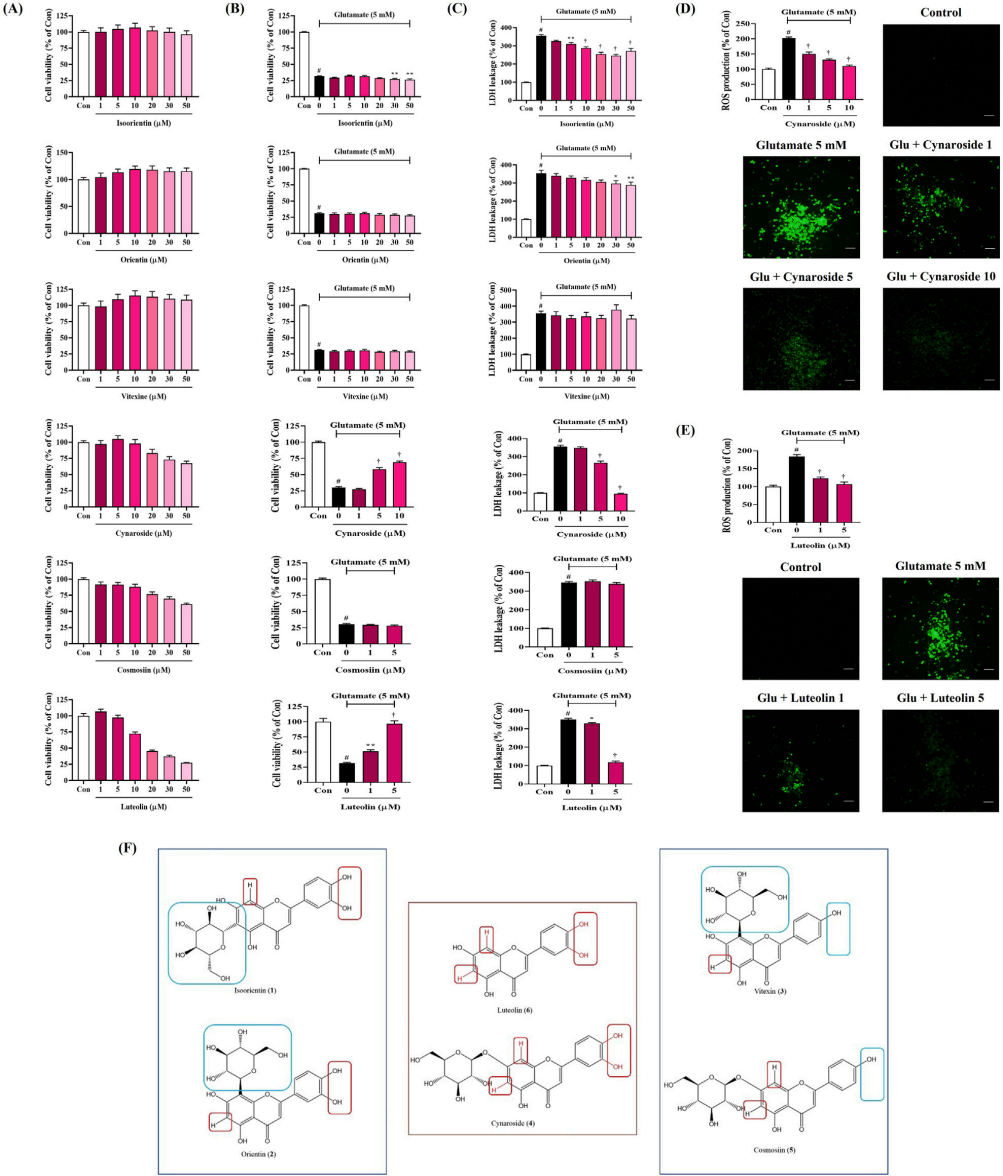
Effects of the main components of Spirodelae Herba ethanol extract (SHE) on glutamate-induced **(A–C)** cytotoxicity and **(D, E)** intracellular reactive oxygen species (ROS) production in HT22 cells and **(F)** structure–activity relationship of the six main components. **(A–E)** Data are expressed as the mean ± standard error of the mean of six independent experiments. **(D, E)** Scale bar = 200 μm. **(F)** Red zone: activity elements; Blue zone: inactivity elements. Con, control; Glu, glutamate; LDH, lactate dehydrogenase. ^#^ *p* < 0.05 (vs. control), ^*^ *p* < 0.05, ^**^ *p* < 0.01, and ^†^ *p* < 0.001 (vs. glutamate).

To explore the effects of the six main components of SHE on glutamate-induced neurotoxicity in HT22 cells, we first investigated the effects of each flavonoid on cell viability. Each flavonoid was treated at 1 μM–50 μM in cultured HT22 cells, and isoorientin, orientin, and vitexin did not show significant cytotoxicity at all concentrations (Figure 8A). In addition, cynaroside did not show cytotoxicity up to 10 μM, and cosmosiin and luteolin did not show cytotoxicity up to 5 μM (Figure 8A). Therefore, in further testing for the neuroprotective activity of each flavonoid, they were used only in the range where they did not show cytotoxicity to completely exclude potential cytotoxicity.

Next, we explored the effects of each flavonoid on glutamate-induced neurotoxicity in HT22 cells. The cell viability of HT22 was reduced by approximately 70% when exposed to glutamate, which was not recovered at all by pretreatment with isoorientin, orientin, vitexin, and cosmosiin (Figure 8B). However, pretreatment with cynaroside and luteolin effectively and concentration-dependently improved the cell viability (Figure 8B).

In addition, LDH leakage, one of the indicators of cell death, was sharply increased by glutamate treatment, which was slightly inhibited by pretreatment with isoorientin and orientin (Figure 8C). Vitexin and cosmosiin did not inhibit LDH leakage at all, whereas cynaroside and luteolin showed excellent inhibitory effects at relatively low concentrations (Figure 8C).

### 3.14 Inhibitory effect of cynaroside and luteolin on ROS generation in HT22 cells exposed to glutamate

We analyzed the effects of cynaroside and luteolin, which showed neurotoxicity-inhibiting effects among the six flavonoids, on ROS accumulation in HT22 cells. As shown in Figures 8D, E, glutamate treatment alone dramatically increased intracellular ROS accumulation. However, this increase was significantly reduced by cynaroside and luteolin pretreatment, and excellent effects were observed at relatively low concentrations (Figures 8D, E).

## 4 Discussion

In the current study, we provide the scientific evidence of the neuroprotective activities of SHE on hippocampal cells exposed to glutamate and the ameliorative activities on SCO-treated cognitive impairment in mice. SHE treatment significantly reversed the decreased viability, increased LDH leakage, increased ROS production, and increased the apoptosis of hippocampal cells exposed to glutamate. SHE exerted antioxidant effects by activating the Nrf-2 mechanism in HT22 hippocampal cells and enhanced BDNF expression by promoting the activation of the ERK/CREB signaling pathway. In addition, SHE inhibited the neuroinflammatory response of microglia BV2 induced by LPS and the activation of the NF-κB/MAPK pathway. In *in vivo* tests, SHE administration significantly improved the memory function and learning ability of mice. In addition, SHE effectively enhanced the activation of the ERK/CREB and PI3K/Akt/GSK-3β signaling pathways in the mouse hippocampus and upregulated BDNF expression.

As is widely known, neurotoxicity and dysregulated neuroinflammatory responses cause damage to the nervous system and are considered to be causes of neurodegenerative diseases (Gulyaeva et al., 2017; Kim et al., 2019; Tan et al., 2013). Therefore, we first investigated whether SHE exerts neuroprotective effects in mouse hippocampal cells exposed to glutamate. SHE pretreatment significantly restored glutamate-induced apoptosis in HT22 cells, thereby exhibiting a high neuroprotective effect (Figure 1). SHE also significantly suppressed LDH leakage, thereby demonstrating a protective effect against neurotoxicity (Figure 1). Furthermore, SHE minimized glutamate-induced oxidative stress stimulation by suppressing ROS accumulation (Figure 1) and effectively inhibited neuronal cell apoptosis (Figure 2). In addition, SHE induced the production of antioxidant enzymes (HO-1 and NQO1) by increasing the nuclear translocation of Nrf-2 in HT22 cells and promoted BDNF expression by activating the ERK/CREB signaling pathway (Figure 2). These results suggest that the neuroprotective effect of SHE is related to the activation of antioxidant mechanisms and the ERK/CREB pathway, which was also well demonstrated in the experiment using MEK inhibitor U0126 (Figure 2). In addition, SHE significantly inhibited microglia-mediated neuroinflammatory responses, and this effect is thought to be because of the regulation of the activation of the NF-κB/MAPK pathway (Figure 3).

Current AD treatment focuses on activating the cholinergic neurotransmitter system. However, such pharmacological treatments, which include donepezil, rivastigmine, and galantamine, induce various side effects, including nausea, vomiting, and headache (Musial et al., 2007; Nwidu et al., 2017). Therefore, we investigated the activity of SHE on cognitive dysfunction induced by cholinergic blockade through behavioral tests such as MWM and PA, exploring natural substances that can inhibit cholinergic blockade. In the MWM test, mice injected with SCO alone showed increased escape distance and latency to reach the platform, indicating impaired memory and learning ability, which established the validity of the animal model. However, 100 mg/kg SHE administration effectively reduced the escape distance and latency, indicating a significant improvement in memory and learning (Figure 4). The PA test results also indicate that 100 mg/kg SHE administration greatly increased the step-through latency of mice, indicating improved working memory compared with the SCO-only group (Figure 4). One peculiarity of the results of the mice behavioral test model is that the efficacy was significantly reduced in the high-dose 200 mg/kg group compared to the significant efficacy of 100 mg/kg SHE. The exact pharmacodynamic reason for the decreased efficacy at 200 mg/kg was not revealed in this study, but the reason can be carefully inferred by applying the concept of hormesis, as discussed in previous studies (Huang et al., 2018). As reported in previous studies on the efficacy of some herbal extracts, there is a potential possibility that a biphasic (nonlinear) dose–response relationship exists in SHE administration in mice (Huang et al., 2018; Jodynis-Liebert and Kujawska, 2020). In addition, it has been previously revealed that high-dose administration can sometimes produce opposing effects due to receptor desensitization, metabolic changes, or activation of compensatory mechanisms (Calabrese and Baldwin, 2003). The cause of the reversal of efficacy depending on the dose of SHE was not revealed in detail in this study, but it could be an interesting research topic to elucidate the reason through pharmacokinetic and pharmacodynamic analyses.

Several research studies have reported that the hippocampus and cortex are the central areas responsible for learning and memory. Furthermore, the structure of pyramidal cells is closely related to cognitive function (Eichenbaum, 2000). We examined the morphology of pyramidal cells and neurons through Nissl staining to analyze the histopathological changes induced by SCO and SHE administration in the hippocampus. As expected, our finding showed that the pathological changes induced by SCO administration in the mouse brain were significantly suppressed by SHE administration, and the number of normal neurons in the cortex and hippocampus was significantly increased (Figure 5). Therefore, based on these results, SHE can effectively improve cognitive impairment induced by SCO.

The ERK/CREB/BDNF is an important signaling pathway related to learning and memory processes. ERK is a well-known protein in the hippocampus and contributes to the stabilization of several neural functions, particularly long-term synaptic plasticity and new memories (Adams and Sweatt, 2002). CREB, a transcription factor activated by ERK, is an important molecule required for learning and memory formation (Tyler et al., 2002). BDNF is an essential factor downstream of phosphorylated CREB that also functions in synaptic plasticity and memory processes. The increased expression of BDNF in the brain can activate synaptogenesis and improve cognitive function (Tyler et al., 2002). The Western blot analysis of the SCO-induced cognitive impairment mouse model was performed to determine whether SHE administration activated the ERK/CREB signaling pathway and promoted BDNF expression to improve cognitive function. Our data indicate that SHE administration not only enhanced BDNF expression levels in mouse hippocampus but also increased ERK and CREB phosphorylation (Figure 5). Another major molecular mechanism correlated with learning and memory is the PI3K/Akt pathway, a multifunctional signaling pathway involved in synaptic plasticity, recognition, memory consolidation, and LTP formation (Peltier et al., 2007). GSK-3β is a signaling molecule downstream of the PI3K/Akt pathway involved in regulating hippocampal neurogenesis and synaptic plasticity (Zhang et al., 2016). Our experimental results clearly indicated that SHE administration upregulated the activation levels of PI3K, Akt, and GSK-3β in mouse hippocampus, thereby activating them (Figure 5). Furthermore, SHE administration can ameliorate cognitive impairment caused by SCO by enhancing neuron survival and synaptic plasticity through the activation of the ERK/CREB/BDNF and PI3K/Akt/GSK-3β signaling pathways in mouse hippocampus.

Recent studies propose that the microbiota–gut–brain axis represents a single system where the gut microbiota and brain interact; thus, improving the gut microbiota could mitigate the development and progression of neurodegenerative diseases (Loh et al., 2024). In our study, SHE administration restored both the α- and β-diversities altered by SCO administration to normal levels (Figure 6). Specifically, we observed significant changes in the microbial composition within the Firmicutes and Bacteroidetes phyla. SCO administration increased Firmicutes and decreased Bacteroidetes, which is consistent with findings in AD mouse models (Brandscheid et al., 2017). These changes induce gut microbiota dysbiosis. Maintaining the balance of the gut microbiota is crucial because dysbiosis affects the secretion of several neurotransmitters and neuromodulators, including serotonin, norepinephrine, dopamine, short-chain fatty acids, γ-aminobutyric acid, and neurotoxic metabolites (Nguyen et al., 2023). Therefore, SHE consumption can alter the specific composition and function of the gut microbiota by stabilizing microbial diversity and reversing dysbiosis.

Next, we conducted phytochemical analysis through HPLC to investigate the relationship between the bioactivity of SHE and its components. We identified six flavonoids as the main components, namely, isoorientin, orientin, vitexin, cynaroside, cosmosiin, and luteolin. In addition, we investigated the neuroprotective effects of the six flavonoids on HT22 cells exposed to glutamate. In the results, cynaroside and luteolin showed neuroprotective effects by inhibiting glutamate-induced cytotoxicity, LDH leakage, and ROS production (Figures 8A–E).

Based on the comparison of the structures and activity results of the six compounds, we can infer the key active functional groups of these flavonoids in the neuroprotective activity test. The hydroxy groups at C-3′ and C-4′ on the B ring are particularly significant (Figure 8F). Compounds **4** and **6**, which contain the hydroxy group at C-3′ and C-4' (red zone), exhibit exceptionally strong activity compared to compounds **3** and **5**, which lack this group (blue zone). Furthermore, substituting other groups at C-6 and C-8 (red zone) on the A ring significantly diminishes activity, as seen with compounds **1** and **2** (blue zone). In summary, the hydrogen atoms at C-6 and C-8 (no substituent), along with the hydroxy groups at C-3′ and C-4′, are essential for activity. Compounds featuring both active groups (compounds **4** and **6**) demonstrate particularly strong activity.

In addition, previous studies have shown that isoorientin improved SCO-induced cognitive dysfunction in mice (Ko et al., 2019). Orientin alleviated cognitive decline and oxidative stress in AD mice (Yu et al., 2015). Luteolin can improve cognitive impairment in mice through neuroprotective activity (Kou et al., 2022; Tan et al., 2020). Therefore, based on the neuroprotective effects of the components identified by HPLC analysis and previous studies, it can be seen that the cognitive enhancement and neuroprotective properties of SHE are closely related to the activities of isoorientin, orientin, cynaroside, and luteolin. These four flavonoids have similar structures, which contain the same luteolin aglycone, and isoorientin, orientin, and cynaroside are glycosides of luteolin, which are converted to luteolin through metabolic processes, suggesting that luteolin and its glycosides are the main active ingredients of SHE.

## 5 Conclusion

In conclusion, SHE significantly improved glutamate-induced oxidative stress and neurotoxicity in HT22 hippocampal cells, which appeared to be related to the activation of the Nrf-2 and ERK/CREB pathways. SHE also suppressed BV2 microglia-mediated neuroinflammation by regulating the NF-κB/MAPK pathway. SHE effectively alleviated SCO-induced cognitive impairment in mice. Specifically, SHE significantly reduced the escape distance and latency of mice in the MWM test and significantly increased the step-through latency in the PA test, mitigating the decreased spatial memory function and working memory of mice caused by SCO. In addition, SHE administration effectively prevented the collapse of normal neurons and pyramidal cells in mouse hippocampus and activated the ERK/CREB/BDNF and PI3K/Akt/GSK-3β mechanisms involved in learning and memory. Microbiome analysis results showed that SHE administration normalized the imbalance in the diversity and composition of the gut microbiota induced by SCO, restoring the healthy balance of the gut microbiota. Therefore, SHE restored both the α- and β-diversities of the gut microbiota to normal levels, and considering the effects of the gut microbiota on cognitive function, the current study results indicate that the cognitive improvement and neuroprotective effects of SHE may be mediated by the normalization of the gut microbiota. Among the key flavonoid components of SHE identified through HPLC, cynaroside and luteolin showed protective effects against neurotoxicity in HT22 cells, and, in addition to these, isoorientin and orientin appear to be related to the beneficial effects of SHE on cognitive and neurological functions. Isoorientin, orientin, and cynaroside are glycosides of luteolin; thus, it can be seen that luteolin and its glycosides are the main active ingredients of SHE. Therefore, in this study, we demonstrate the potential application of SHE as a therapeutic agent for preventing and treating neurotoxicity and neuroinflammation-related cognitive impairment and neurodegenerative diseases.

## Data Availability

The original contributions presented in the study are publicly available. This data can be found here: https://www.ncbi.nlm.nih.gov/bioproject/PRJNA1307222.
